# A Case Read at the Meeting of the Organon Society of Boston, December 28th

**Published:** 1890-01

**Authors:** Wm. P. Wesselhœft

**Affiliations:** Boston, Mass.


### A CASE READ AT THE MEETING OF THE ORGANON SOCIETY, OF BOSTON, DECEMBER 28th.

By Wm. P. Wesselhœft, M. D., Boston, Mass.

To illustrate three facts, first: The difficulty of getting a good record by the first examination with many of our patients, who come to be relieved of certain symptoms which to them are paramount and the most annoying at times, and if not absolutely denying the physician's right to further inquiries, they answer questions vaguely or as if they had no relation whatever to what they came "to be doctored for."

Secondly: To show how in the course of the treatment of a chronic disease, the reappearance of old forgotten symptoms should be estimated, which ought to have formed part of the first examination.

Thirdly: That it is right to regard the most recent symptoms in a case as the indicative ones for the selection of the remedy.

H. B. A., æt. twenty-seven. Blonde, thin, active. For a year troubled with diarrhœa. Always has a loose, watery stool at seven P. M. A second stool may follow any time during the day —early evening, forenoon, or afternoon. The stools are very urgent, often nothing but a little sputter with much flatus; is obliged to run to the closet as soon as he feels the desire, as he has but little power to retain stool.

Much rumbling of wind in abdomen after going to bed. Usually awakens an hour after going to bed with palpitation of heart; after passing flatus goes to sleep and rests easily the remainder of the night. At night he can pass flatus with confidence, which he could not do during the day. All the flatus he passes is hot.

Free discharge of prostatic fluid after stool. Constant sensation of soreness in lower abdomen, over os pubis, not sensitive to pressure. Tongue clean, appetite very good.

He avers that he has been well all his life up to a year ago. When a boy had tinea ciliaris.

Now, what bothered this young man more than anything was the discharge of prostatic fluid after the stool, and that is what he came to be "doctored for." We all know that such a solitary symptom will give us no indication for a remedy, and if I had known as much as I do now about this symptom thirty years ago it would have saved me much trouble and often anxiety. In every instance I should have made this symptom a secondary and not a primary indication, no matter what the wishes of my patients might have been. Instead of trying all the remedies enumerated under the head of discharge of prostatic fluid during stool, I should have worked at other more important features of the case. But how often is the young physician misled by the desire to put an end to the symptom which most disturbs the patient's mind, and especially if he comes with a diagnosis already concocted by some celebrity which aids and abets the fears of the patient?

The diarrhœa with the characteristic weakness of the sphincter, which would not allow him at any time, except in the night, to pass flatus, the flatus always being hot when passed; the clean tongue and good appetite led me to give him a dose of Aloes.$^{cm}$

In a fortnight he came back with the following story:

One formed stool a day for the last ten days. No urgency. Passes flatus with confidence and is not hot. Has slept well every night, no palpitation. Very little prostatic fluid has passed.

*Reappearance of sick headaches, of which he had two violent ones during the fortnight.* These have been absent for over a year and were treated by Bromo-caffeine.

Now, consider for a moment my astonishment when my patient told me that he had always suffered from sick headaches up to the time his other troubles commenced! I gave Sac. Lac.

A fortnight later came the following report:

Stools have remained perfectly normal. No discharge of prostatic fluid for two weeks. Soreness in lower abdomen over region of bladder entirely gone. During the fortnight has had four severe headaches with nausea but no vomiting. Gets very faint at stomach every morning about ten o'clock—another old symptom which accompanied his former sick headaches. Just forty days after the dose of Aloes he received a dose of Sulph.$^{cm}$

Three weeks later he reports:

No headache to speak of, one or two attempts at one, but not severe enough to keep him from work. His stools remain normal. Is troubled a little with flatulence which has easy and confident egress. Has gained four pounds during the last three weeks. Is discharged cured.

Inferences: Aloes cleared up the case for Sulphur. Sulphur would have cured his headaches when he first had them; and all the subsequent symptoms, owing to suppression of psoric headaches, were due solely to a masking of a case suited homœopathically and originally for Sulphur.

